# Synergistic antitumor efficacy of a decorin-carrying oncolytic adenovirus combined with chemotherapy in pancreatic cancer

**DOI:** 10.3389/fonc.2026.1702601

**Published:** 2026-03-17

**Authors:** Jing Chen, Wei Ping Tian, Xiao Bai Wei, Xi Wei Zhang, Hong Yuan Gao

**Affiliations:** 1 Department of Oncology, Jing’an Central Hospital, Shanghai, China; 2 Interventional Radiology Department of Gongli Hospital in Pudong New Area, Shanghai, China

**Keywords:** chemotherapy, decorin, immune regulation, oncolytic adenovirus, pancreatic cancer

## Abstract

Advances in gene engineering technology have led to the increasing clinical application of oncolytic adenovirus-targeted cancer therapy. Previous studies have demonstrated that oncolytic adenoviruses carrying decorin exhibit significant efficacy in inhibiting the growth of solid tumors. Additionally, these viruses have been shown to participate in the assembly of the tumor extracellular matrix (ECM) by expressing decorin in a subcutaneous renal cell carcinoma tumor model, thereby improving the tumor immune microenvironment. Pancreatic ductal adenocarcinoma (PDAC) is one of the leading causes of cancer-related deaths worldwide, characterized by its insidious onset, limited treatment options, and poor long-term survival rates. The matrix surrounding pancreatic tumors is believed to be a key mediator of disease progression through its direct effects on cancer cells and indirect effects on the tumor immune microenvironment. In this study, we constructed a subcutaneous tumor model of pancreatic cancer and applied a combination of Decorin-carrying oncolytic adenoviruses and chemotherapy for *in vivo* and *in vitro* experiments. The results showed that the combination of the two further enhanced tumor-killing effects. *In vivo* experiments further confirmed that OAV-Decorin can promote IFN-γ expression, suppress TGF-β expression, improve the tumor-suppressive microenvironment, and ultimately achieve an antitumor effect.

## Introduction

Pancreatic cancer is a relatively common malignant tumor of the digestive tract, characterized by extremely high malignancy and poor prognosis. It is projected to become the second leading cause of cancer-related deaths by 2030 (1). Approximately 80% of pancreatic cancer patients present with advanced or metastatic disease, making them ineligible for curative surgery (2). As a result, combination chemotherapy has become the primary treatment modality. Nevertheless, after combination chemotherapy, the median survival time of patients can only be prolonged by 2–4 months (3), accompanied by significant toxic side effects. Related studies have shown that the occurrence, development, and outcome of pancreatic cancer are related to the surrounding immune microenvironment. Immune cells, inflammatory cells, fibroblasts, the cell matrix, and related signaling molecules in the immune microenvironment play an important role in the occurrence and development of tumors (4, 5). On the other hand, dense stroma exists outside pancreatic tumor tissues, penetrating and surrounding the tumor tissues, thereby impairing the delivery and penetration of clinical drugs and weakening their therapeutic efficacy (6). Therefore, new treatment methods are urgently needed to address these limitations.

Oncolytic virus (OV) can be engineered to selectively infect tumor cells, lysis and kill tumor cells, release viral particles, infect neighboring tumor cells, spread within tumor tissues, and simultaneously activate the host’s antiviral and antitumor immune responses, thereby preventing tumor metastasis and recurrence (7). Decorin is a low-molecular-weight proteoglycan rich in leucine, recognized as a tumor suppressor gene. It can bind to various growth factors and cytokines to regulate cell growth and tissue restructuring (8, 9), and can also bind to and inactivate transforming growth factor β (TGF-β) and inhibit vascular endothelial growth factor (VEGF) expression. Through activating the EGFR/MAP kinase/p21 signaling pathway to inhibit tumor cell proliferation and metastasis. It plays a crucial role in tumor development, angiogenesis, and metastasis (10). Decorin expression mediated by oncolytic adenovirus effectively inhibits tumor progression and metastasis in various tumor models (11).

In the aim of enhancing the efficacy of chemotherapy drugs and the role of oncolytic adenoviruses in the tumor immunosuppressive microenvironment, we combined oncolytic adenovirus armed with decorin (OAV-Decorin) with chemotherapy and tested it on pancreatic cancer cells *in vivo* and *in vitro*. Our findings revealed that OAV-Decorin directly lyses tumor cells, facilitating the penetration of chemotherapy drugs into cells. Additionally, the decorin carried by OAV enhances the inflammatory immune state, improves tumor tissue permeability, and suppresses the tumor TGF-β signaling pathway. When combined with chemotherapy, this synergistically enhances antitumor activity. These discoveries may provide a better strategy for addressing the limited tumor penetration capacity and drug resistance associated with chemotherapy in solid tumors.

## Materials and methods

### Cell culture

Human pancreatic cancer cells PANC-1, human pancreatic cancer cells Capan-2, and human pancreatic cancer cells BXPC-3 were purchased from Wuhan Qisai Bio-Technology Company. PANC-1 cells were cultured in high-glucose DMEM medium supplemented with 10% fetal bovine serum (FBS) and 1% penicillin/streptomycin dual antibiotics. Capan-2 cells were cultured in McCoy’s 5A medium supplemented with 10% FBS and 1% dual antibiotics. BXPC-3 cells were cultured in RPMI-1640 medium supplemented with 10% FBS and 1% penicillin-streptomycin. All cell lines were maintained in a 37 °C incubator with 5% CO_2_ and ≥95% humidity. For thawing, rapidly thaw cryovials at 37 °C. Centrifuge the cell suspension, resuspend in the appropriate medium, and plate. Change the medium after 24 hours. When confluence reaches 80-90%, all the above cell lines can be passaged for subsequent culture.

### Adenoviruses construction

The shuttle plasmids pCMV-decorin and pCMV-EGFP were co-transfected with the adenovirus cytoskeleton plasmid pBHGE-Lox (Microbix Biosystems) into HEK-293 cells. Transfection was performed using Lipfectamine 2000 (Invitrogen) according to the manufacturer’s instructions. Viral plaques appeared within 9–14 days, yielding recombinant OAV-Decorin and OAV. Virus plaques were then picked for expansion. Finally, HEK293 cells were infected with adenovirus Ad-decorin and Ad-EGFP, respectively. When half the cells had detached, the supernatant was collected. Viral supernatants were purified using an adenovirus purification kit, and titers were determined by the tissue culture infectious dose 50 (TCID50) assay. The purified viruses were stored at -80 °C for future use.

### CCK-8 proliferation experiment

Initially, OAV and OAV-Decorin were used to infect pancreatic cancer cells BXCP-3, Capan-2, and PANC-1 by MOI = 0, 5, 20, and 50, respectively. After 24, 48, and 72 hours, cell proliferation was recorded and analyzed, and pancreatic cancer cell proliferation was measured using the CCK-8 method. Next, to further screen out the most suitable chemotherapy drug concentration, the above three experimental cells were added with gemcitabine at concentrations of 5 μmol/L and 25 μmol/L, incubated for 48 hours, and then CCK-8 was used to measure the effect of different concentrations of gemcitabine on pancreatic cancer cell proliferation. Lastly, in order to verify whether there is a synergistic effect between OAV-Decorin and chemotherapy on the inhibition of pancreatic cancer cell proliferation, we selected the most suitable chemotherapy drug concentrations screened in the experiment, and infected pancreatic cancer cells BXCP-3, Capan-2, and PANC-1, followed by the addition of 25 μmol/L gemcitabine. After 24 hours, the CCK-8 method was used to evaluate the effect of OAV-Decorin combined with chemotherapy on pancreatic cancer cell proliferation. Survival rate was calculated as follows: survival rate = (OD value of the experimental group - OD value of the blank group)/(OD value of the control group - OD value of the blank group) × 100%.

### Transwell tests cell migration

OAV and OAV-Decorin were used to infect pancreatic cancer cells BXCP-3, Capan-2, and PANC-1 at MOI = 0, 5, 20, and 50, respectively. After 24 hours, Transwell was used to observe the effects of OAV and OAV-Decorin at different MOI on the migration of pancreatic cancer cells. Subsequently, the experimental cells were cultured for 48 hours, then the culture dishes were removed, washed with PBS buffer, fixed with 4% polyformaldehyde at room temperature for 30 minutes, washed three times with PBS buffer, stained with 0.5% crystal violet staining solution, photographed under an inverted microscope on the non-cell-seeded side, and cell counts were performed using Image J software.

### Flow cytometry to detect cell apoptosis

OAV and OAV-Decorin were used to infect pancreatic cancer cells BXCP-3, Capan-2, and PANC-1 at multiplicity of infection (MOI) values of 5, 20, and 50, respectively; the cells were then co-cultured with 25 μmol/L gemcitabine for 48 hours before harvesting cells in the logarithmic growth phase (adherent cells digested with 0.25% trypsin, suspension cells directly centrifuged). The harvested cells were centrifuged at 1000 rpm for 5 minutes, the supernatant was discarded, and the cells were gently washed twice with pre-chilled PBS (each wash followed by centrifugation at 1000 rpm for 5 minutes); after discarding all PBS, 100 μL of pre-chilled 1× Binding Buffer was added to the cell pellet to gently resuspend the cells. Subsequently, the Annexin V-FITC/PI apoptosis detection kit (BD Biosciences, San Jose, CA, USA) was used following the manufacturer’s instructions: the mixture was gently mixed and incubated at room temperature (20-25 °C) in the dark for 15 minutes, and immediately after incubation, 400 μL of pre-chilled 1× Binding Buffer was added to the tube, mixed gently, and flow cytometry (Beckman, USA) was used to measure the apoptosis rate.

### Western blot

Proteins were extracted from cells in each experimental group and their concentrations were measured. Forty micrograms of total protein were loaded onto SDS-PAGE gels for electrophoresis. After electrophoresis, equal amounts of protein were transferred from the gel to PVDF membranes. The membranes were incubated at room temperature for 1 hour in blocking buffer (TBS containing 0.1% Tween 20 supplemented with 5% skim milk powder). Subsequently, samples were incubated overnight at 4 °C with Primary antibodies targeting mouse anti-E1A (Santa Cruz, Cat. No. sc-58658. Clone M58) or rabbit anti-β-actin (Tiande Yue, Beijing, Cat. No. TDY051).After thorough washing, membranes were incubated at room temperature for 1 hour with horseradish peroxidase (HRP)-labeled IgG secondary antibodies: HRP-Goat anti-Mouse (Aspen, Cat. No. AS1106) and HRP-Goat anti-Rabbit (Aspen, Durban, South Africa Cat. No. AS1107). The blotted membranes were visualized using a chemiluminescent detector.

### ELISA experiment

BXCP-3, Capan-2, and PANC-1 cells were infected with OAV and OAV-Decorin at a multiplicity of infection (MOI) of 20. After 24 hours of culture in 10% FBS medium, the culture medium was removed, and the cells were continued to be cultured in serum-free medium for another 48 hours. Subsequently, the supernatant from each well was collected and centrifuged at 4000 rpm for 5 minutes. The expression of TGF-β1 and Decorin was then tested by ELISA. In the *in vivo* experiment, we also used ELISA to test the expression of IFN-γ in mouse serum and IFN-γ, TGF-β, and Decorin in tumors. After euthanizing the mice, blood was collected from the heart, and the blood samples were left to stand at room temperature for 30 minutes. then centrifuged at 3000 rpm for 10 minutes, and the supernatant was collected. Then, tumor tissue was minced using ophthalmic scissors, weighed, and 0.1 g of tissue was placed into an EP tube. Add 1 mL of tissue lysis buffer containing 1% PMSF, and homogenize the tissue using a tissue homogenizer. After homogenization, transfer the homogenized liquid to a new EP tube. Centrifuge at 13,000 rpm at 4 °C for 10 minutes, collect the supernatant as a sample, and use ELISA to examine the expression of TGF-β1, IFN-γ, and Decorin. All experimental procedures were performed according to the manufacturer’s instructions for the TGF-β ELISA kit (Reed Biotech, China. Product code: RE10013), the Decorin (ELK Biotechnology, China. Product code: ELK3013), or IFN-γ (ELK Biotechnology, China. Product code: ELK1132) kits.

### Animals experiment

Male NTG mice aged 6–8 weeks (n = 30) were purchased from Wuhan Besei Model Biotechnology Co., Ltd, this immunodeficiency mouse strain (animal line code: C202) was generated by knocking out the IL2rg gene using CRISPR/Cas9 technology in NOD-SCID mice with Prkdc gene mutations. Animal welfare and laboratory procedures were strictly conducted in accordance with the Guidelines for the Care and Use of Laboratory Animals. Additionally, all animal experiments were approved by the Animal Welfare and Ethics Committee of the Wuhan Besei Model Biotechnology Center. NTG mice were subcutaneously injected with pancreatic cancer PANC-1 cells (5 × 10^6^/mouse) in the right flank to establish a pancreatic cancer xenograft model. When the tumor diameter reached 3–5 mm, NTG mice were randomly divided into six groups (PBS group, OAV group, OAV-Decorin group, chemotherapy group, OAV-Decorin+chemotherapy group, OAV+chemotherapy group), with 5 mice per group. However, 12 mice failed to develop tumors within 2 weeks post-injection, meeting the criterion of a tumor volume ≥50 mm³ for successful tumor formation. Consequently, 18 mice with established tumors were retained and regrouped into the original 6 groups (3 mice per group) for subsequent treatment. Intratumoral injections were administered with 1×10^9^ PFU of OAV, OAV-Decorin, or an equal volume of PBS, every other day for a total of three doses. In the chemotherapy groups, gemcitabine was administered at a concentration of 50 mg/kg via intravenous injection every three days. Starting from the day of treatment, the longest and shortest diameters of the transplanted tumors were measured. Measurements were taken every 4 days throughout the treatment period, and the tumor volumes were calculated. The average values were then plotted to construct tumor growth curves. On day 43 of treatment, the mice were euthanized, the tumors were excised, and fixed in 4% paraformaldehyde.

### Immunohistochemistry staining analysis

Tumor tissue was fixed in 10% formaldehyde, embedded in paraffin, and sectioned into 3mm thick slices. After dewaxing, tumor sections were treated with anti-Decorin primary antibody (Wuhan Sanying Biotechnology Co., Ltd. Product code:14667-1-AP) and incubated overnight at 4 °C. The following day, sections were washed three times with PBS, followed by addition of HRP-labeled secondary antibody (Aspen BioSciences, Inc., USA. Product code: AS1107) and incubation at room temperature. After secondary antibody incubation, tissue sections were counterstained with hematoxylin and finally examined under light microscopy.

### Statistical analysis

Data are expressed as the mean ± standard deviation (SD). Experimental data were analyzed using SPSS 16.0 statistical software. Statistical significance was defined as a P value < 0.05 (*p < 0.05; **p < 0.01; ***p <0.001; **** p < 0.0001.).

### Euthanasia of animals

After the experiment concluded, the preparation and calibration of euthanasia equipment were first completed, including carbon dioxide (CO_2_) cylinders, precision gas control valves, and a sealed euthanasia chamber; after that, the experimental mice were gently placed into the sealed chamber, the door was confirmed to be fully closed to prevent escape or gas leakage, and the main valve of the CO_2_ cylinder and the gas control valve were opened—initially, CO_2_ was slowly introduced at a flow rate replacing 10%–30% of the chamber volume per minute, and the gas concentration was gradually increased until complete saturation was reached to minimize stress response in the mice; once the mice consistently showed loss of consciousness and absence of spontaneous body movements, the gas flow could be moderately increased but was strictly limited to a maximum of 0.5 kPa to prevent additional suffering, and throughout the procedure, vital signs had to be continuously monitored until the complete cessation of body movements, termination of respiration, and dilated pupils were confirmed; after the preliminary confirmation of death, the main valve of the CO_2_ cylinder was immediately closed, observation was continued for 2–3 minutes, and during this period, death had to be reconfirmed through two core indicators: first, the complete cessation of thoracic movement was observed to confirm respiratory arrest, and second, the mice’s toes were gently pinched to confirm the absence of any pain reflex; following successful reconfirmation, the mice’s carcass were safely removed, and the subsequent disposal were completed according to standardized procedures for laboratory animal waste.

## Results

### Evaluation of the therapeutic efficacy and characteristics of decorin-carrying oncolytic adenoviruses against pancreatic cancer cells *in vitro*

**Figure 1:**
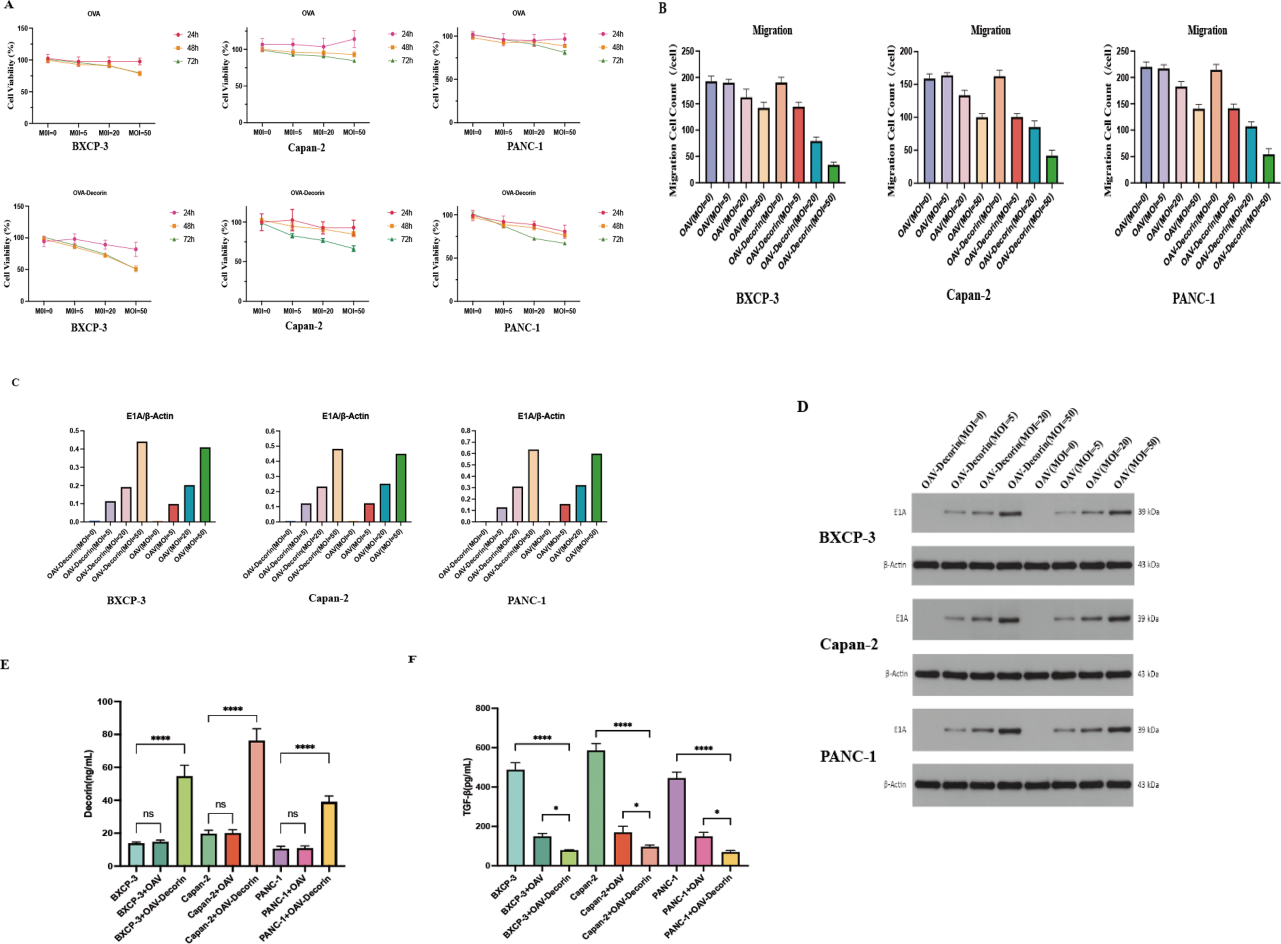
The oncolytic viruses expressing decorin (OAV-Decorin) inhibit the proliferation and migration of pancreatic cancer cells *in vitro*. **(A)** BXCP-3, Capan-2,and PANC-1 cells were infected with OAV-DEC and OAV respectively at a various MOIs (0, 5, 20, and 50) for 48, 72, or 96 h, Cell viability was measured using the CCK-8 assay, Relative cell viability was calculated; **(B)** BXCP-3, Capan-2,and PANC-1cells were infected with OAV-DEC and OAV respectively at various MOIs (0, 5, 20, and 50) for 24 h, Transwell Assay measured the number of migrating cells.;BXCP-3, Capan-2,and PANC-1 cells were infected with OAV-DEC and OAV respectively at various MOIs (0, 5, 20, and 50); 48 h later, the key adenoviral E1A protein was confirmed by Western blot. β-Actin was used as a loading control. **(C, D)** The three pancreatic cancer cell lines were respectively infected at the indicated MOIs of OAV-DEC and OAV. After 48 hours, supernatants were collected, and the concentration of Decorin and TGF-β were analyzed by ELISA assay **(E, F)**. ^**^p < 0.01, ^****^p < 0.0001.

We have applied an engineered oncolytic adenovirus expressing decorin N, named OAV-E1-Decorin (OAV-DEC), using gene recombination technology with the selective oncolytic adenovirus ZD55 as a vector. Initially, the therapeutic efficacy of OAV-Decorin on different pancreatic cancer cell lines was evaluated using CCK-8 and Transwell assays. Pancreatic cancer cell lines BXCP-3, Capan-2, and PANC-1 with MOI values of 0, 5, 20, and 50, and recorded cell viability and migratory cell counts. The results showed that as the MOI increased, the inhibitory effect of OAV-decorin on pancreatic cancer cell proliferation and migration became more pronounced (**Figures 1A, B**). Additionally, Western blot (WB) analysis was used to detect E1A gene expression mediated by OAV-decorin. The results indicated that the expression levels of adenovirus E1A in the three pancreatic cancer cell lines increased in a dose-dependent manner (**Figures 1C, D**). Compared with the control group, the expression level of Decorin in OAV-decorin infected pancreatic cancer cells was higher and could be efficiently released, as confirmed by ELISA (**Figure 1E**). Next, TGF-β expression in infected pancreatic cancer cells was examined. As shown in **Figures 1F**, TGF-β expression levels in OAV-decorin treated pancreatic cancer cells decreased gradually with increasing dose. These observations indicate that adenoviruses carrying decorin can efficiently infect pancreatic cancer cells, inhibit their proliferation and migration, produce and secrete high levels of functional decorin, suppress TGF-β expression, and improve tumor immune suppression.

### Gemcitabine inhibits pancreatic cancer cell viability

**Figure 2:**
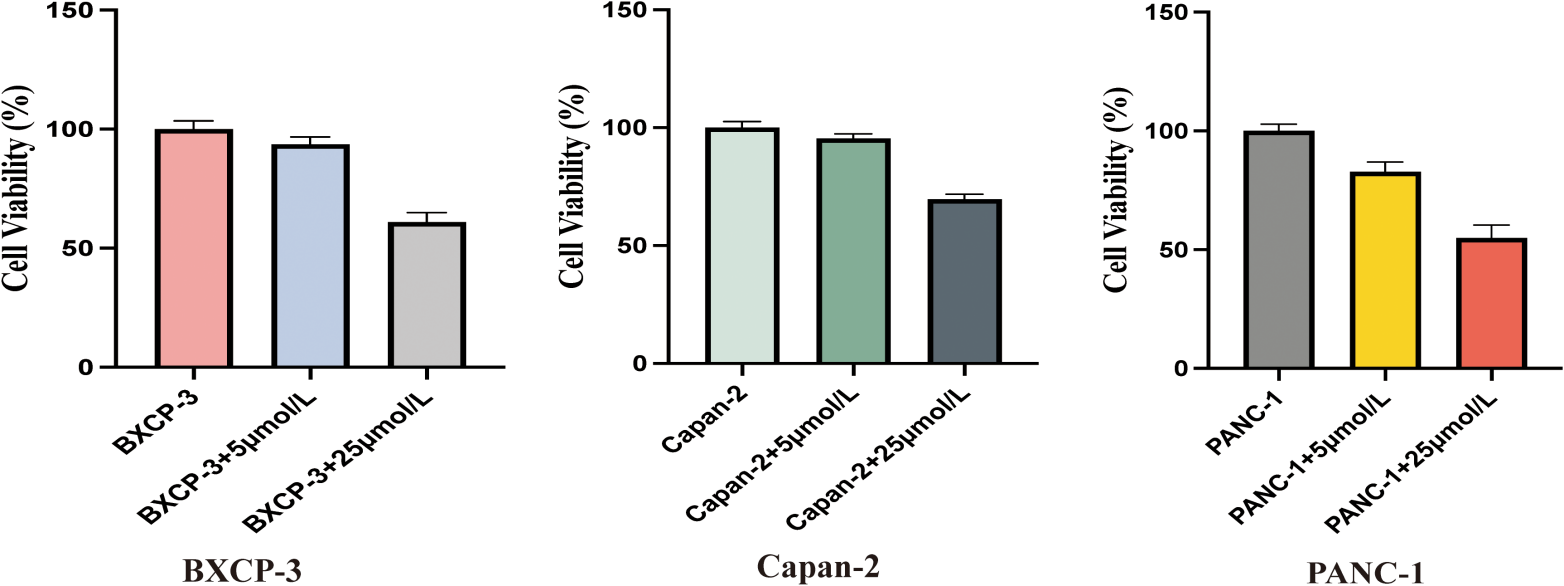
Gemcitabine inhibits pancreatic cancer cells proliferation in a density-dependent manner. BXCP-3, Capan-2, and PANC-1 were treated with gemcitabine at concentrations of 5 μmol/L and 25 μmol/L, respectively. After 48 hours, the CCK-8 method was used to test the cell viability.

Despite the fact that gemcitabine monotherapy is currently one of the main treatment options for pancreatic cancer, in order to obtain more accurate results for this experiment and select the optimal drug concentration, pancreatic cancer cell lines BXCP-3, Capan-2, and PANC-1 were treated with gemcitabine at concentrations of 5 μmol/L and 25 μmol/L, respectively. After 48 hours, the CCK-8 method was used to assess the effect of varying gemcitabine concentrations on the proliferation of pancreatic cancer cells. The results indicate that the inhibitory effect on pancreatic cancer cells increases with higher concentrations of gemcitabine (**Figure 2**).

### OAV-decorin combination chemotherapy has stronger antitumor effects than monotherapy *in vitro*

**Figure 3:**
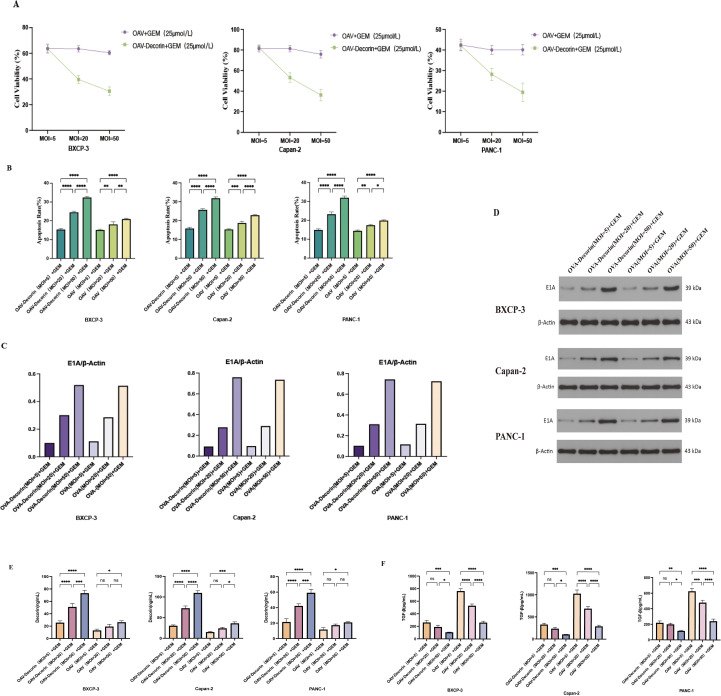
OAV-Decorin enhances antitumor activity in combination chemotherapy *in vitro*, and down-regulates TGF-β protein expression to improve the tumor immunosuppressive microenvironment. **(A)** BXCP-3, Capan-2, and PANC-1 cells were infected with OAV-DEC and OAV, respectively, at various MOIs (5, 20, and 50) for 48 h, Then, 25 μmol/L gemcitabine was added and incubate for 24 hours. Cell viability was measured using the CCK-8 assay. Relative cell viability was calculated. **(B)** BXCP-3, Capan-2, and PANC-1 cells were infected with OAV-DEC and OAV, respectively, at various MOIs (5, 20, and 50), then a concentration of 25 μmol/L of gemcitabine was added and cultured for 48 hours. Flow cytometry was used to detect cell apoptosis. **(C, D)** BXCP-3, Capan-2, and PANC-1 cells were treated in the same method as mentioned above. Total cell lysates were harvested for the Western blot assay to determine E1A protein expression. β-Actin was used as a loading control. **(E, F)** BXCP-3, Capan-2, and PANC-1 cells were infected with OAV-DEC and OAV, respectively, at various MOIs (5, 20, and 50). Then, a concentration of 25 μmol/L of gemcitabine was added and cultured for 48 hours. Decorin and TGF-β expression were determined by ELISA according to the manufacturer’s protocol for ELISA kits. ^*^p < 0.05, ^**^p < 0.01, ^***^p < 0.001, ^****^p < 0.0001.

We aimed to test the antitumor effects of the combination of oncolytic adenovirus OAV-DEC and chemotherapy *in vitro*. OAV and OAV-Decorin were used to infect pancreatic cancer cells BXCP-3, Capan-2, and PANC-1 at MOIs of 5, 20, and 50, respectively, followed by the addition of gemcitabine at a concentration of 25 μmol/L. After 48 hours, 25 μmol/L gemcitabine was added, followed by incubation for 24 hours. The effect of OAV-Decorin combined with chemotherapy on the proliferation of pancreatic cancer cells was evaluated using the CCK-8 method. As shown in **Figures 3A**, with increasing MOI values, the inhibitory effect of OAV-Decorin combined chemotherapy on pancreatic cancer cell growth became more pronounced, particularly in PANC-1 cells. Subsequently, flow cytometry was used to analyze the effect of OAV-Decorin combined chemotherapy on pancreatic cancer cell apoptosis. The results showed that as the MOI increased, the pro-apoptotic effect of OAV-Decorin combined chemotherapy on pancreatic cancer cells was enhanced (**Figure 3B**). The flow cytometry apoptosis graph is shown in (**Supplementary Figure 1**). The E1A gene is the first viral gene transcribed after adenovirus transfects tumor cells and plays a key role in viral replication. In order to investigate whether the expression of OVS-decorin transcription protein was affected after combined chemotherapy, WB was used to analyze the expression of E1A protein in cells. MOI = 5, 20, 50 OAV and OAV-Decorin were transfected into pancreatic cancer cells BXCP-3, Capan-2, and PANC-1, followed by the addition of 25 μmol/L gemcitabine. After 24 hours of co-culture, Western Blot was used to observe the expression of E1A protein in the cells. The results showed that with the increase in the number of infections, the expression of E1A also increased, indicating that chemotherapy drugs promote the replication of OAV-Decorin in pancreatic cancer cells (**Figures 3C, D**). Finally, ELISA was used to examine the effect of OAV-Decorin combined with chemotherapy on the expression of decorin and TGF-β secreted in the culture supernatant of pancreatic cancer cells infected with pancreatic cancer cells. The results showed that after combined treatment, with increasing MOI, the expression of TGF-β decreased, while the expression of decorin increased (**Figures 3E, F**). The above results indicate that the combination of OAV-DEC and chemotherapy can effectively inhibit the growth of pancreatic cancer cells *in vitro*, and this combination therapy is promising.

### OAV-decorin and chemotherapy combination therapy had a stronger antitumor effect than single-agent chemotherapy in the treatment of pancreatic cancer *in vivo*

**Figure 4:**
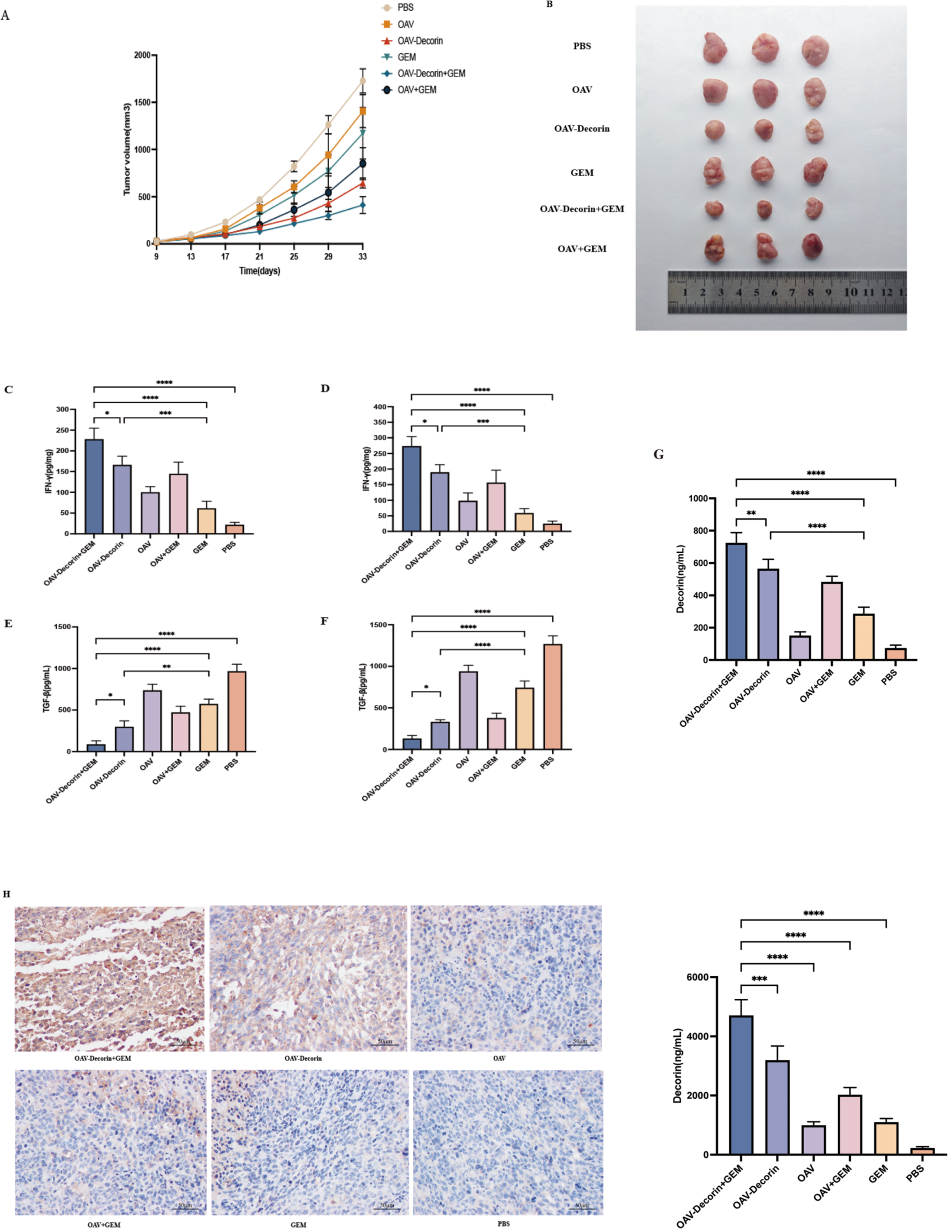
Combination of OAV-Decorin and chemotherapy improved the antitumor effect *in vivo*. NTG mice were subcutaneously inoculated with PANC-1cells (n = 3 per group). **(A, B)** Tumor growth is expressed as the mean tumor volume ± SD was displayed. The production of IFN-γ in serum **(C)** or intratumor **(D)** and TGF-β in serum **(E)** or intratumor **(F)** from euthanized mice was analyzed by ELISA. Decorin **(G)** expression in tumor tissues was detected by ELISA. **(H)** The tumors were removed from the euthanized mice, and the immune infiltrate was evaluated by immunohistochemical staining according to the distribution of Decorin. Scale bar, 20 mm. *p < 0.05, **p < 0.01, ***p < 0.001, ****p < 0.0001.

Virus therapy and chemotherapy are currently considered promising treatment strategies in the field of cancer treatment. In the aim of investigating the *in vivo* efficacy of OAV-Decorin combined with chemotherapy for pancreatic cancer, we established a subcutaneous human pancreatic cancer cell transplant model in NTG mice using the PANC-1 cell line. When the tumor diameter reached 3–5 mm, 18NTG mice were randomly divided into six groups (PBS group, OAV group, OAV-Decorin group, chemotherapy group, OAV-Decorin + chemotherapy group, and OAV + chemotherapy group), with 3 mice in each group. Treatment was initiated. Mice in different experimental groups were administered OAV or OAV-Decorin via intratumoral injection. In the combination chemotherapy group, gemcitabine was administered via intravenous injection immediately after the oncolytic virus injection, while the chemotherapy group received gemcitabine at a concentration of 50 mg/kg via intravenous injection. A control group was established using an equal volume of physiological saline (PBS). Compared with the PBS-treated group, tumor volume growth was inhibited in the OAV, OAV-Decorin, OAV-Decorin + chemotherapy, OAV combined chemotherapy, and chemotherapy groups. Among these, OAV-Decorin combined chemotherapy significantly inhibited tumor volume growth (**Figures 4A, B**). At the end of the experiment, NSG mice were euthanized, and blood was collected from the heart, while tumor homogenate and supernatant were collected. We analyzed the expression levels of interferon-γ (IFN-γ) in the serum of euthanized mice using an ELISA assay. Compared with the chemotherapy group, the OAV group, or the OAV + chemotherapy group, the IFN-γ expression levels were significantly higher in the OAV-Decorin + chemotherapy group (**Figure 4C**). We also assessed IFN-γ production in the tumor lysate. The results were consistent with the above findings, showing significantly elevated IFN-γ levels in the OAV-Decorin + chemotherapy group (**Figure 4D**). In addition, our results also showed that OAV-Decorin combined chemotherapy significantly increased Decorin expression in pancreatic cancer cells within serum and tumor tissues while simultaneously suppressing TGF-β expression in both serum and tumor tissues compared to the chemotherapy-only group. (**Figures 4E, F**), indicating that the secretion of Decorin in tumors promotes inflammatory responses in the tumor microenvironment and blocks the activity of TGF-β. In addition, we evaluated Decorin expression using immunohistochemical staining. The results showed that compared with the chemotherapy group, the OAV group, or the OAV + chemotherapy group, the number of Decorin-positive staining in tumor tissues was increased in the OAV-Decorin and OAV-Decorin + chemotherapy groups, with the most significant increase observed in the OAV-Decorin + chemotherapy group (**Figures 4G, H**). These findings collectively indicate that OAV-Decorin combined with chemotherapy enhances immune responses and improves tumor control in the treatment of pancreatic cancer.

## Discussion

Oncolytic adenovirus therapy for tumors is an emerging treatment modality that offers cancer patients additional therapeutic options. Researchers have engineered these viruses through genetic recombination to create safer, more targeted oncolytic adenoviruses that directly lyse tumor cells and modulate the tumor microenvironment (TME) to activate the body’s anti-tumor immune response (12). Pancreatic tumor tissues are surrounded by a dense matrix that penetrates and envelops the tumor, impairing the delivery and penetration of clinical drugs. This matrix is considered a key mediator in disease progression, as it directly affects cancer cells and indirectly influences the tumor immune microenvironment, making it difficult for chemotherapy drugs to efficiently penetrate the extracellular matrix and reach the tumor site to exert their effects (13, 14). Therefore, the key to gene-modified oncolytic adenovirus therapy for pancreatic cancer lies in its ability to degrade the extracellular matrix of tumor cells, increase the permeability of therapeutic drugs, promote their infiltration into tumor cells, and at the same time have an oncolytic effect, reverse the tumor immune suppressive microenvironment, enhance anti-tumor capacity, and thereby obtain better anti-tumor activity.

We have developed a combination therapy using an oncolytic adenovirus (OAV) carrying decorin and gemcitabine. Decorin is an effective pan-cancer tumor suppressor that not only interacts with receptor tyrosine kinases but also acts as a pan-receptor tyrosine kinase inhibitor, thereby inhibiting carcinogenic signaling (10). At the same time, within tumors, decorin can also bind to multiple high-affinity RTKs and exhibit multiple tumor suppression functions, including growth inhibition, tumor cell autophagy, and angiogenesis inhibition (15). Therefore, we used genetic recombination technology to produce this type of oncolytic adenovirus. It is hoped that the modified oncolytic adenovirus can alter the composition of the ECM in tumor tissues, enhance tissue permeability, further enhance the infiltration of OAV and chemotherapy drugs into tumor tissues, promote oncolysis, and improve immunosuppression (16). Cell experiments have shown that OAV-Decorin combined with chemotherapy has a significant antitumor effect on pancreatic cancer cells, inhibiting cell proliferation and promoting apoptosis. In addition, in the subcutaneous tumor transplantation model of NTG mice, the experimental results showed that the tumor growth rate of the OAV-Decorin + chemotherapy group was significantly lower than that of the chemotherapy alone group, indicating that OAV-Decorin combined with chemotherapy can effectively inhibit tumor growth. The combined use of oncolytic virus therapy and chemotherapy is expected to become part of future multimodal treatment strategies.

IFN-γ is the most important cytokine in adaptive antitumor immunity. It can directly act on tumor cells to induce cancer cell senescence and promote antitumor immune responses (17, 18). TGF-β is a multifunctional cytokine that promotes angiogenesis in tumor tissues, suppresses the immune system to enable tumor cells to evade killing, and alters the adhesion of tumor cells to surrounding cells, thereby promoting their growth and metastasis (19). Spearman correlation analysis revealed a positive correlation between TGF-β protein and pancreatic cancer, suggesting that TGF-β protein expression may play a crucial role in pancreatic cancer development (20). We found that in mouse models, ELISA results indicated that the antitumor activity of OAV-Decorin was manifested by increased levels of interferon-γ (IFN-γ) protein in tumor tissues, while the levels of transforming growth factor-β (TGF-β) in tumor tissues were significantly lower than those in the OAV-treated group. This suggests that oncolytic adenovirus-mediated Decorin can improve the tumor immunosuppressive microenvironment. Additionally, the IFN-γ expression levels in the OAV-Decorin + chemotherapy group were significantly higher than those in the chemotherapy-only group, and the expression trend of IFN-γ in mouse tumors was consistent with that in serum. TGF-β levels were lower than those in the OAV-Decorin monotherapy group and the chemotherapy-only group, confirming that the combination of the two therapies enhances antitumor immune function.

In conclusion, we demonstrated that OAV-Decorin combined with chemotherapy exhibits synergistic antitumor effects both *in vitro* and *in vivo*. These data lay the foundation for further clinical studies of combination therapy for pancreatic cancer.

In this study, we demonstrated the synergistic antitumor effect of combinational therapy using an oncolytic adenovirus carrying decorin and chemotherapy for pancreatic cancer, though limitations of the model should be noted. The dense tumor stroma in pancreatic cancer constitutes a core barrier to clinical chemotherapy. Composed of activated pancreatic stellate cells (PSCs), high collagen content, and extracellular matrix (ECM), it mediates resistance through both physical obstruction and signaling regulation (21, 22). The NST immunodeficient mouse xenograft model used in this study supports tumor growth and viral replication but fails to fully replicate the complex matrix unique to human pancreatic cancer. This is due to species differences in the activation characteristics of mouse and human PSCs and the proportion of ECM components, as well as the absence of a functional host immune system to regulate matrix remodeling (23). Therefore, the efficacy of the combined therapy in modulating the matrix within the NST model may be underestimated due to the simplified matrix environment. Future validation using genetically engineered mouse models or patient-derived xenograft (PDX) models is warranted to assess the penetration of this synergistic therapeutic strategy into clinically relevant complex matrices and its ability to reverse drug resistance. Such validation would provide more reliable experimental evidence for its clinical translation.

## Data Availability

The original contributions presented in the study are included in the article/**Supplementary Material**. Further inquiries can be directed to the corresponding author.
