# Supplementary figures and images for: Synergistic antitumor efficacy of a decorin-carrying oncolytic adenovirus combined with chemotherapy in pancreatic cancer
Source: Front Oncol. 2026 Mar 17;16:1702601. doi: 10.3389/fonc.2026.1702601 (PMC13035519; doi:10.3389/fonc.2026.1702601)

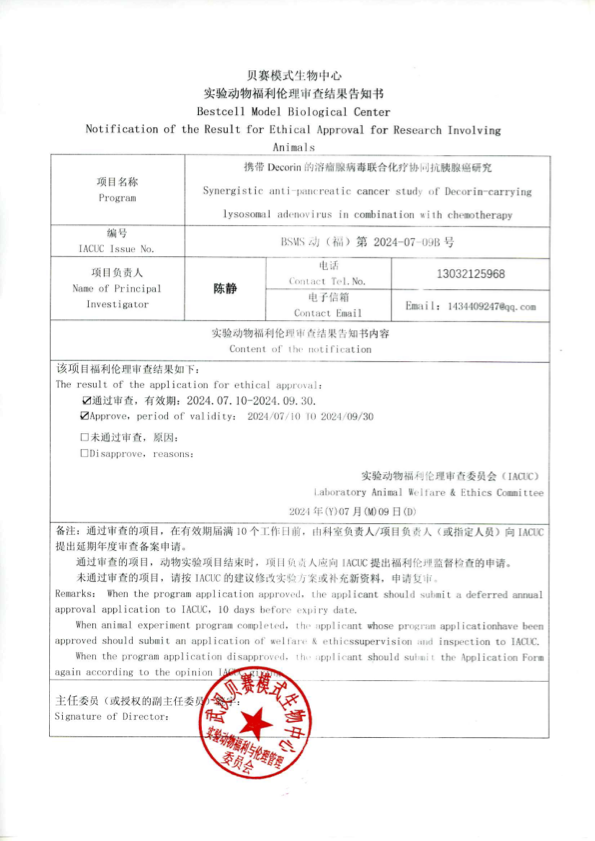

Supplement: Supplementary file 1 [file Image1.tiff]

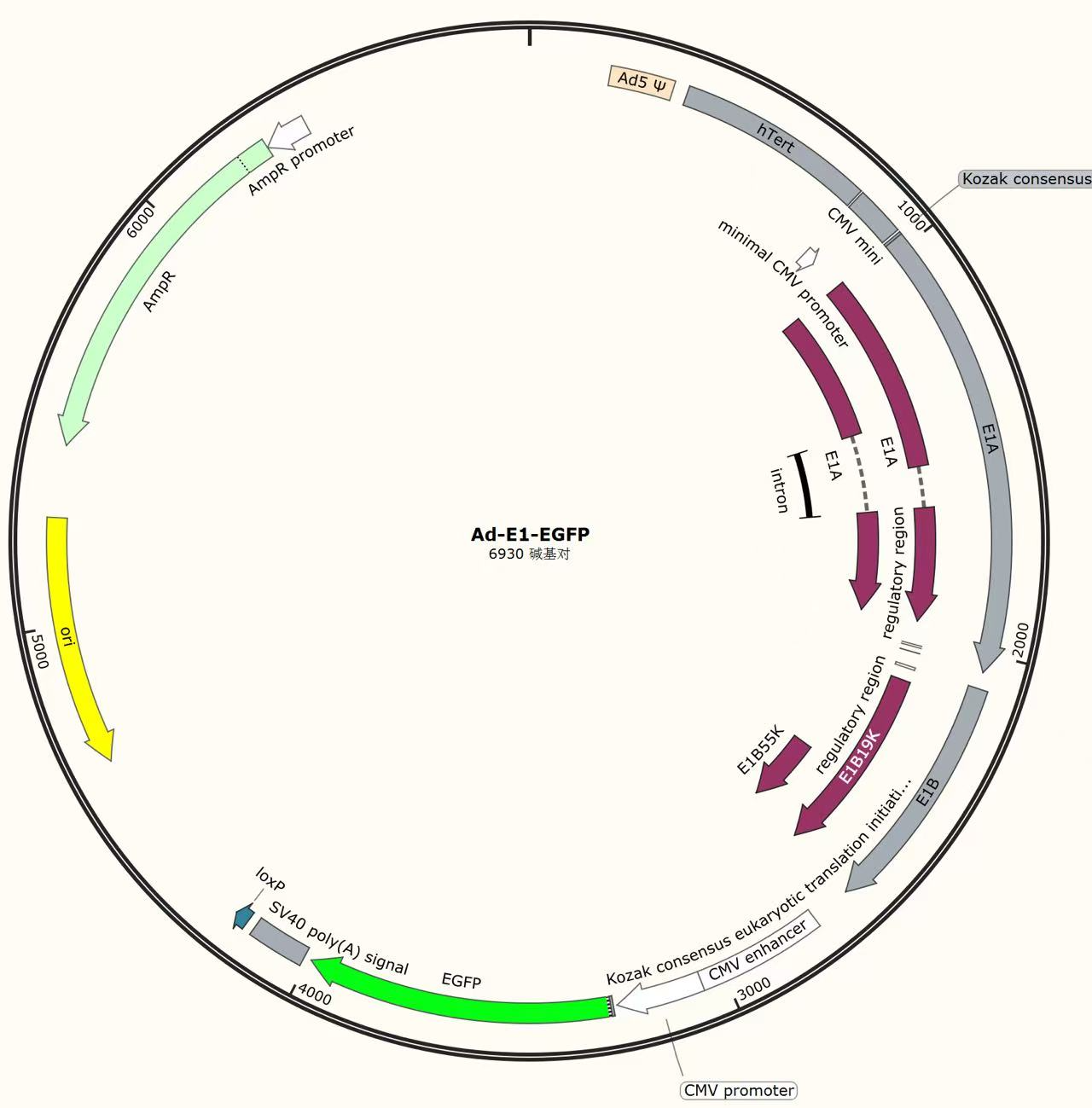

Supplement: Supplementary file 2 [file Image2.tiff]

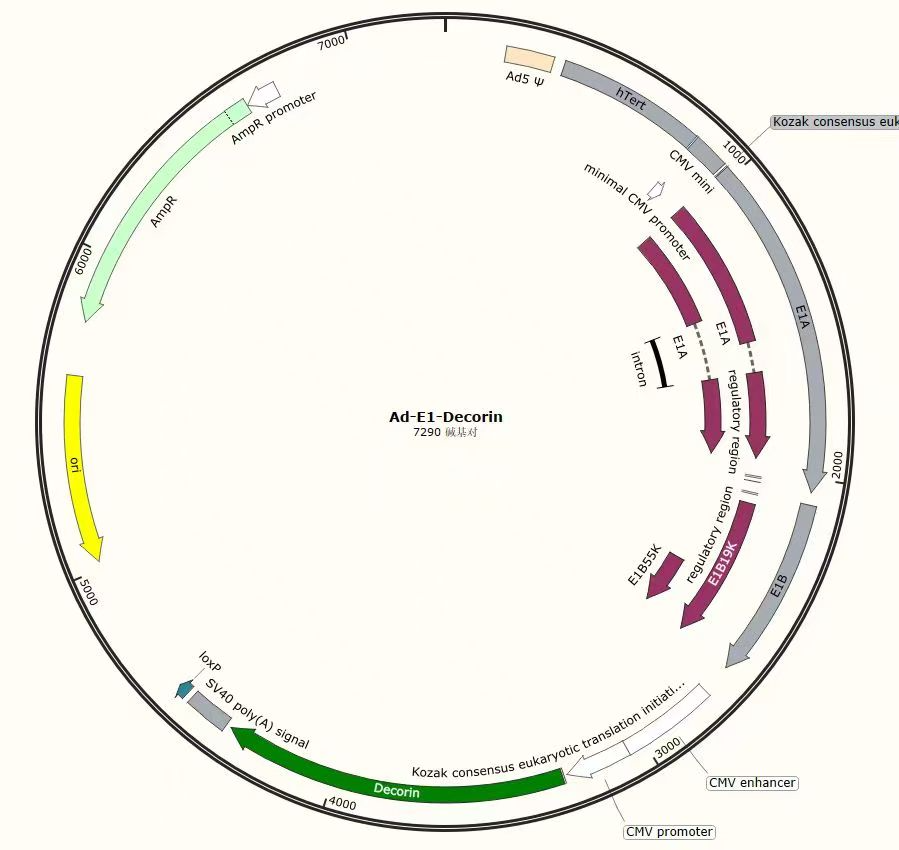

Supplement: Supplementary file 3 [file Image3.tiff]

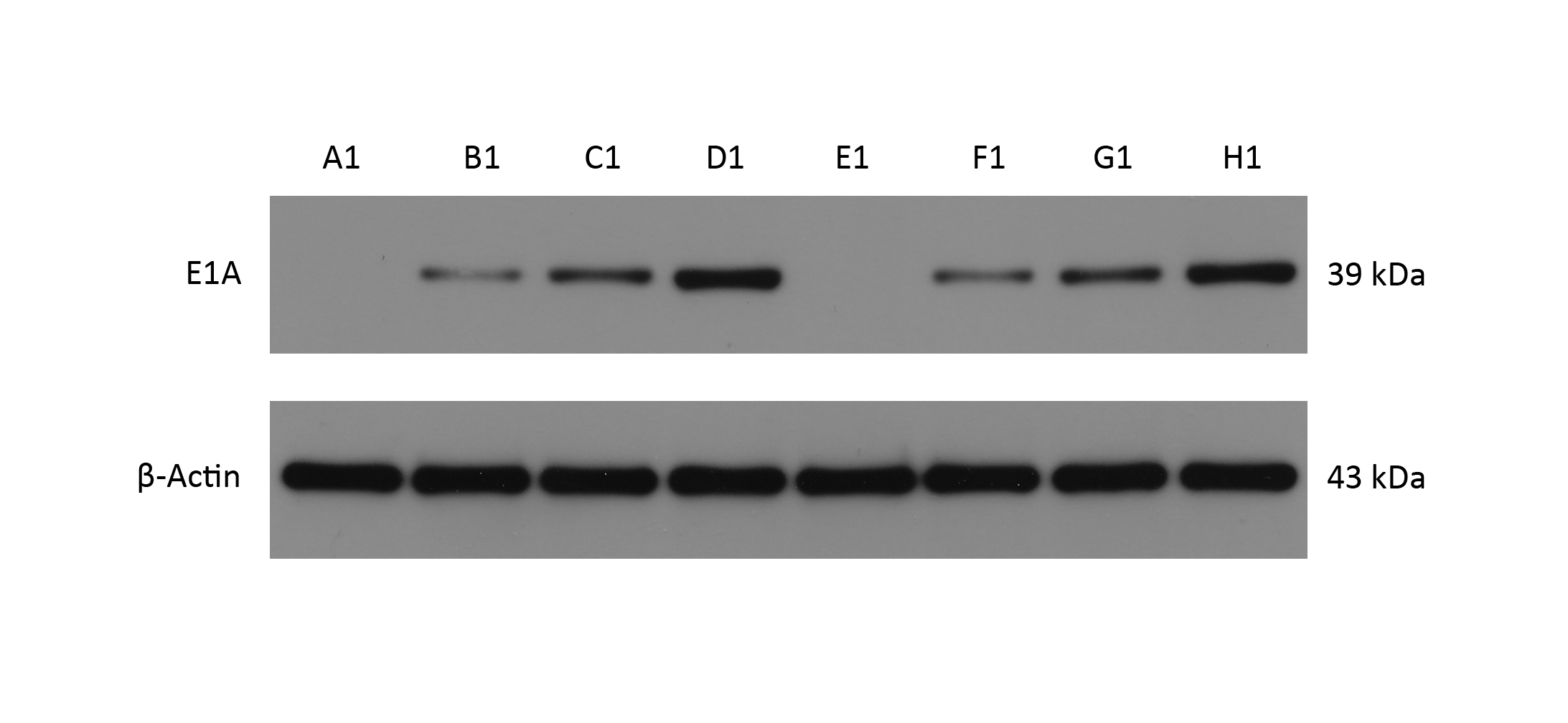

Supplement: Supplementary file 4 [file Image4.tif]

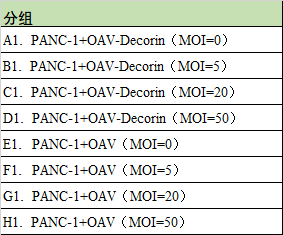

Supplement: Supplementary file 5 [file Image5.tiff]

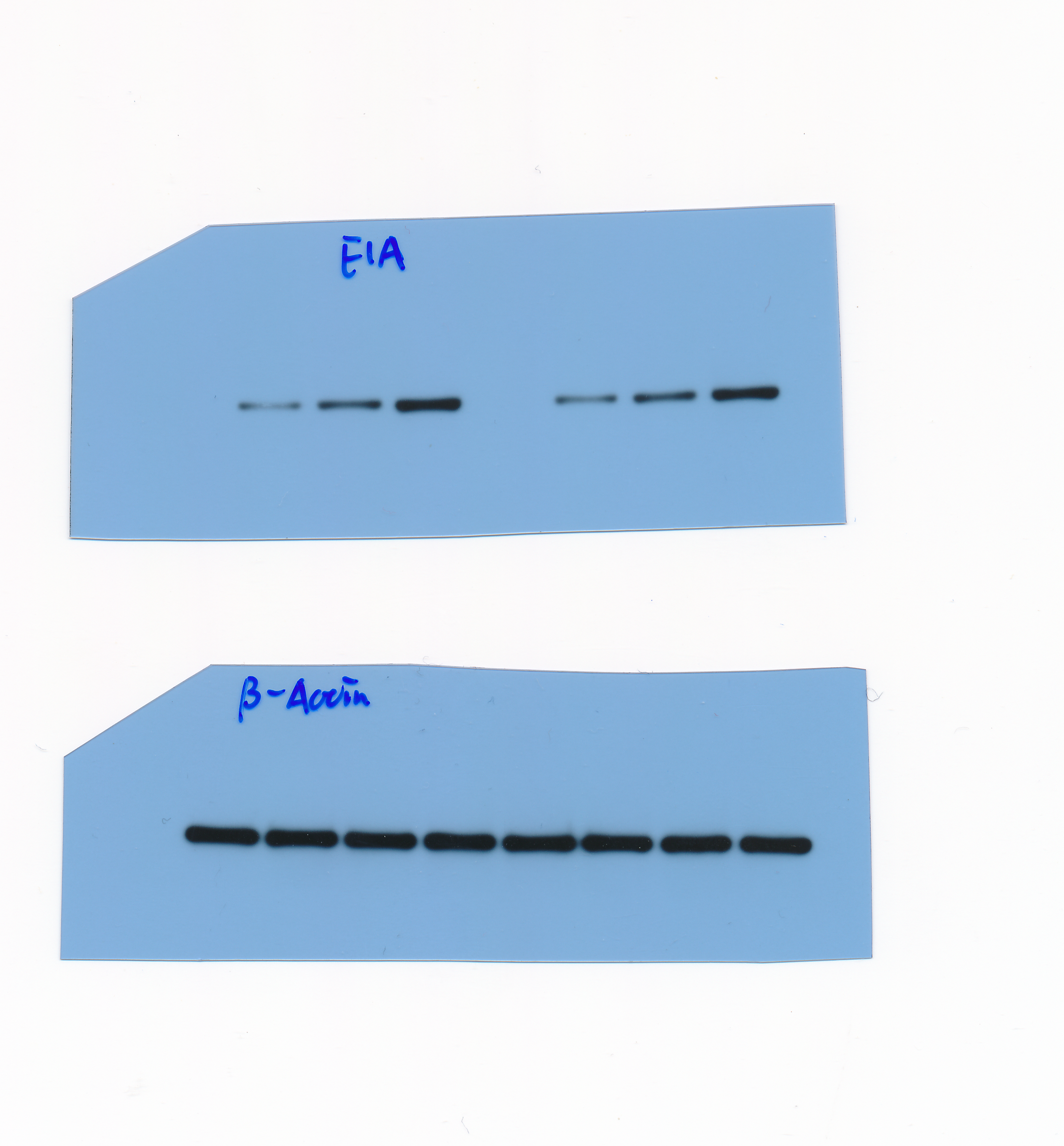

Supplement: Supplementary file 6 [file Image6.tif]

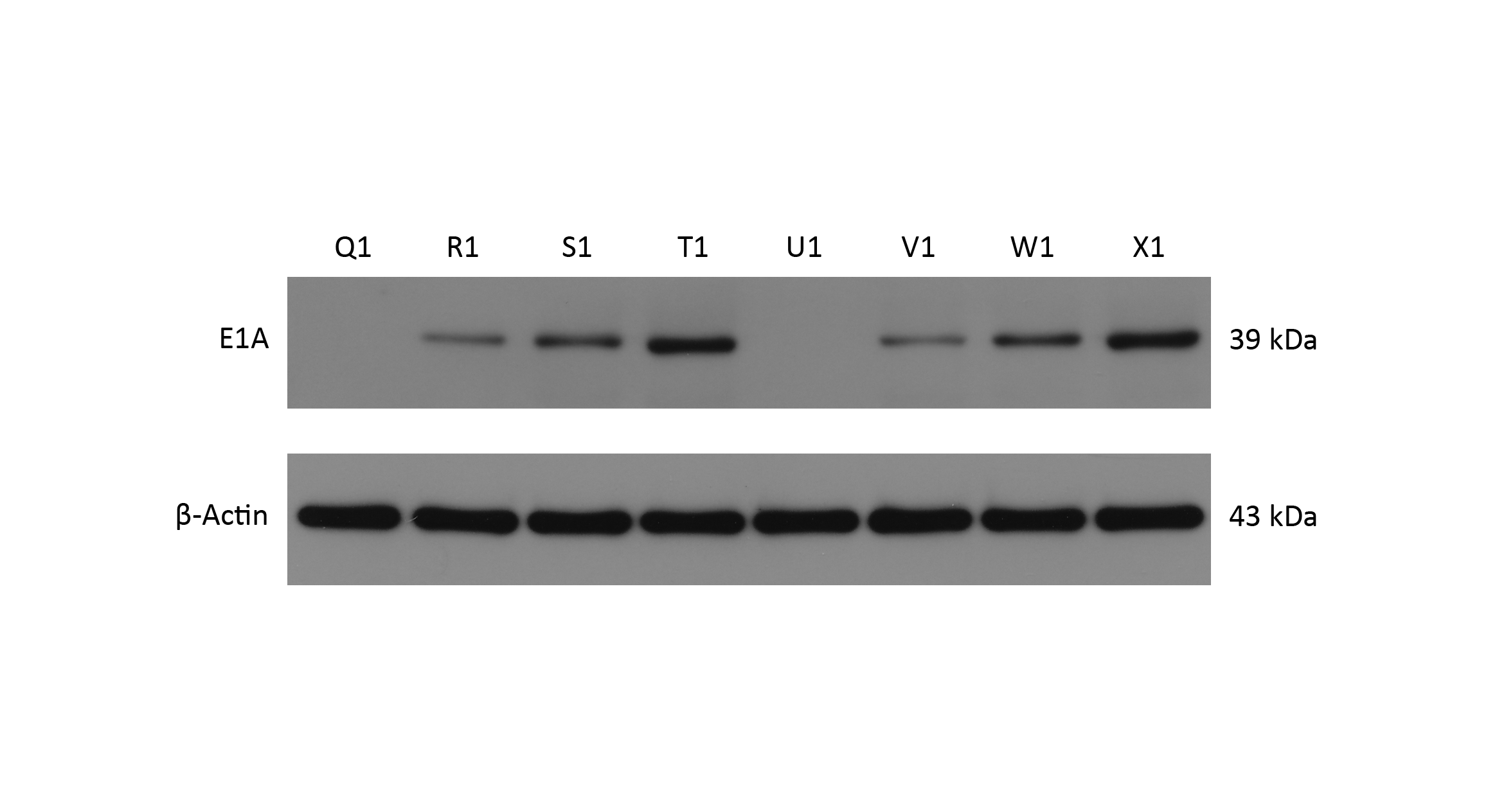

Supplement: Supplementary file 7 [file Image7.tif]

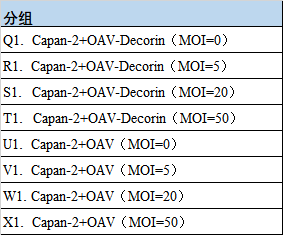

Supplement: Supplementary file 8 [file Image8.tiff]

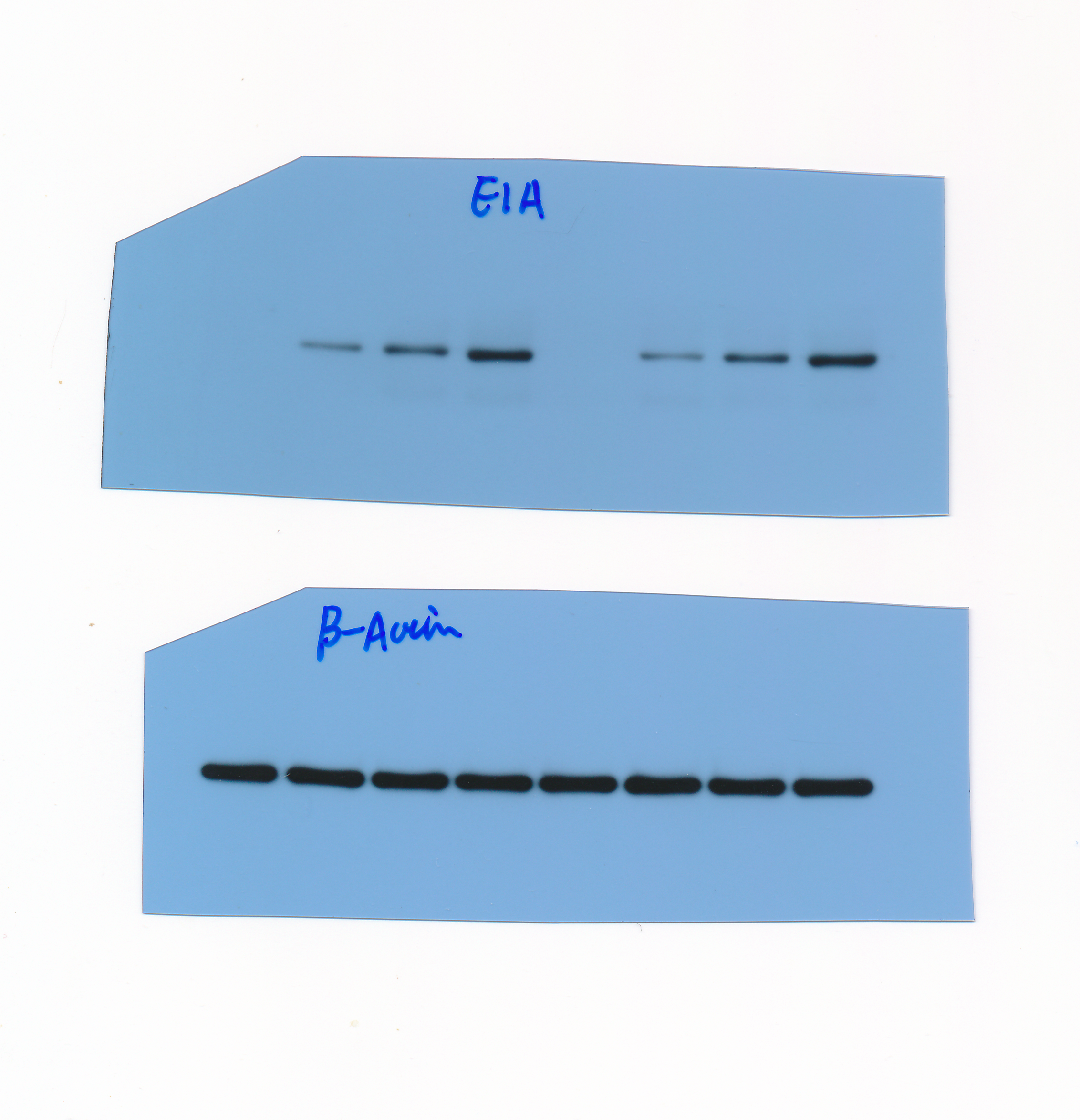

Supplement: Supplementary file 9 [file Image9.tif]

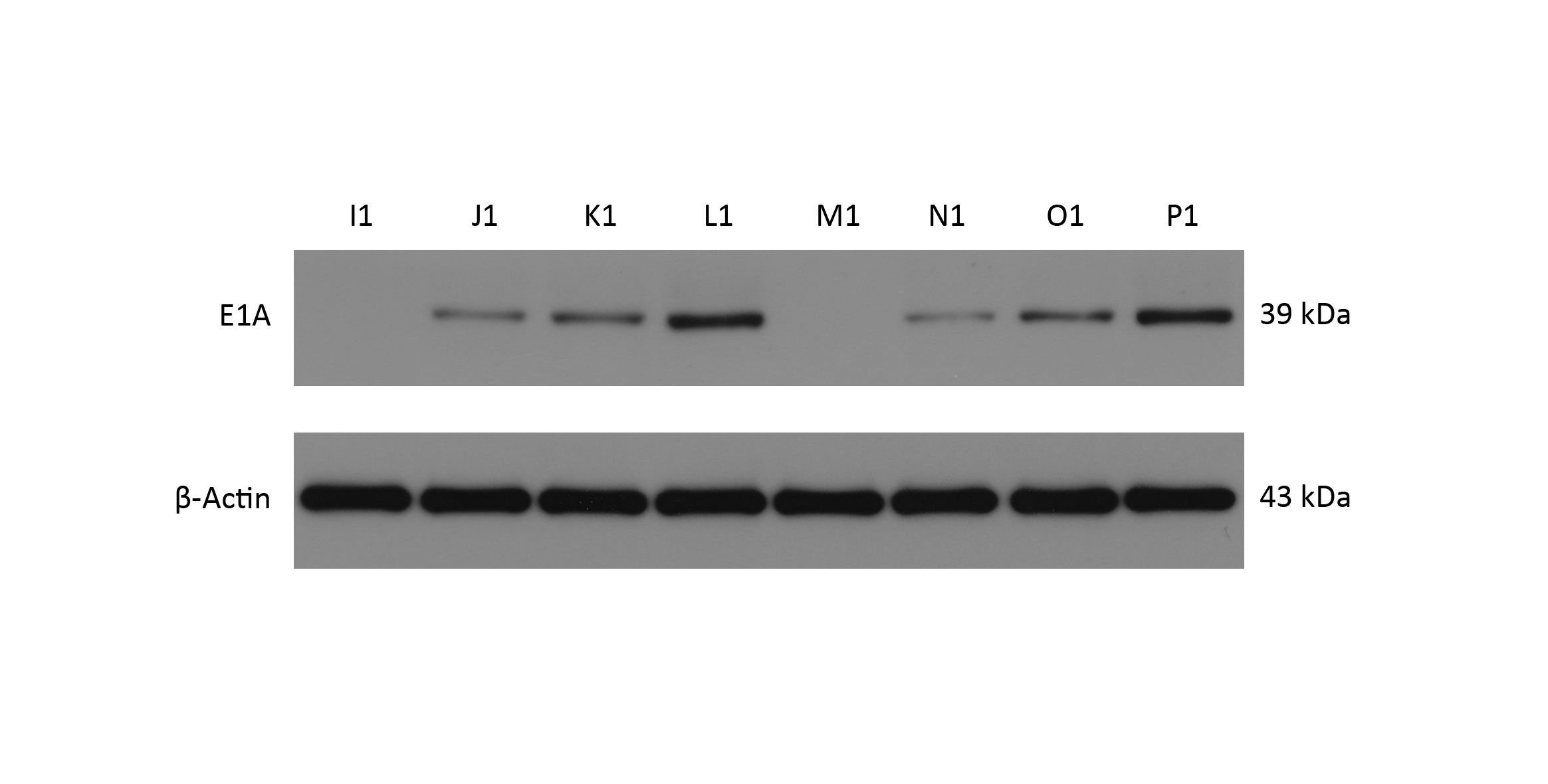

Supplement: Supplementary file 10 [file Image10.tif]

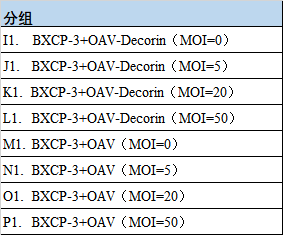

Supplement: Supplementary file 11 [file Image11.tiff]

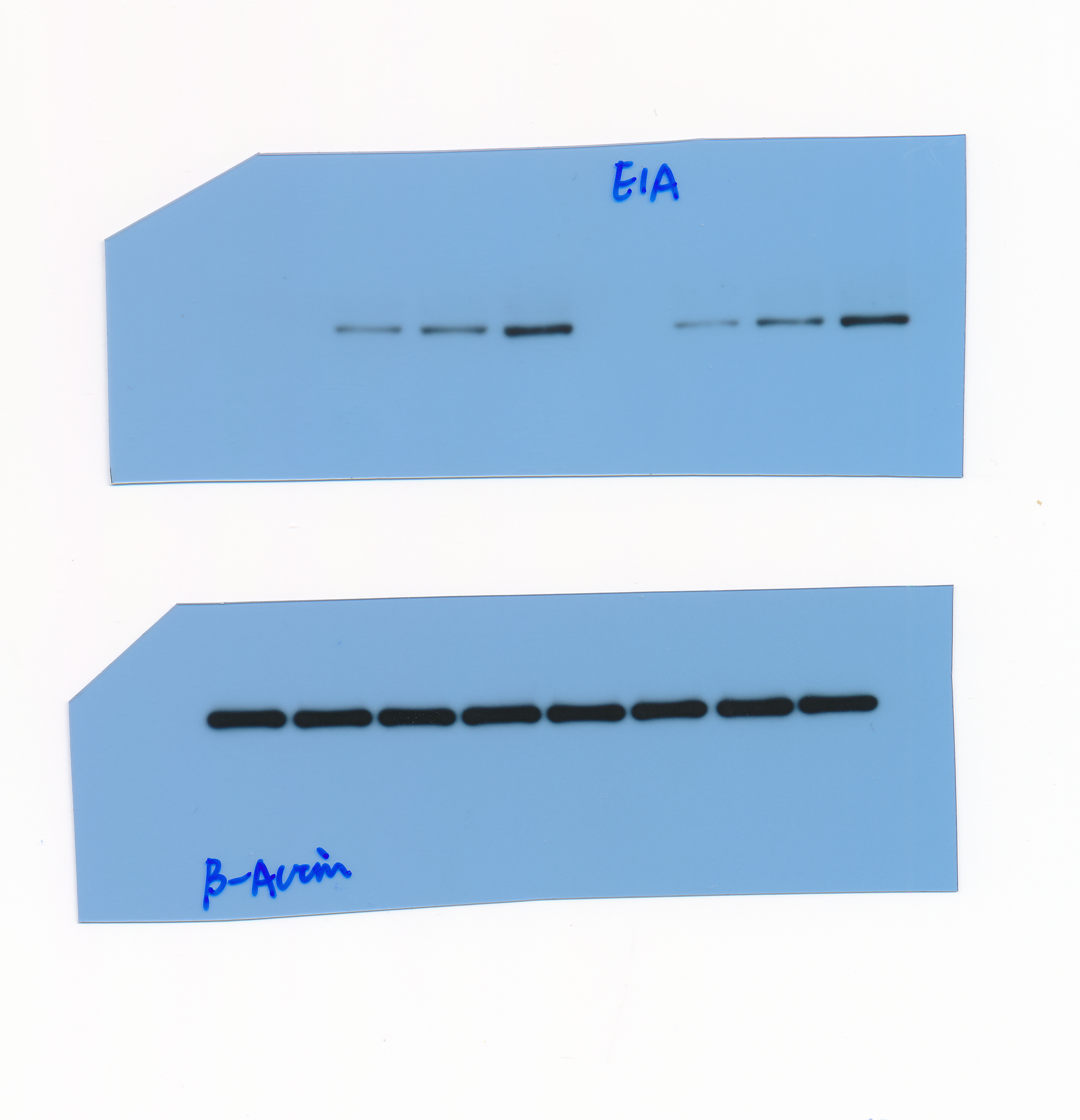

Supplement: Supplementary file 12 [file Image12.tif]

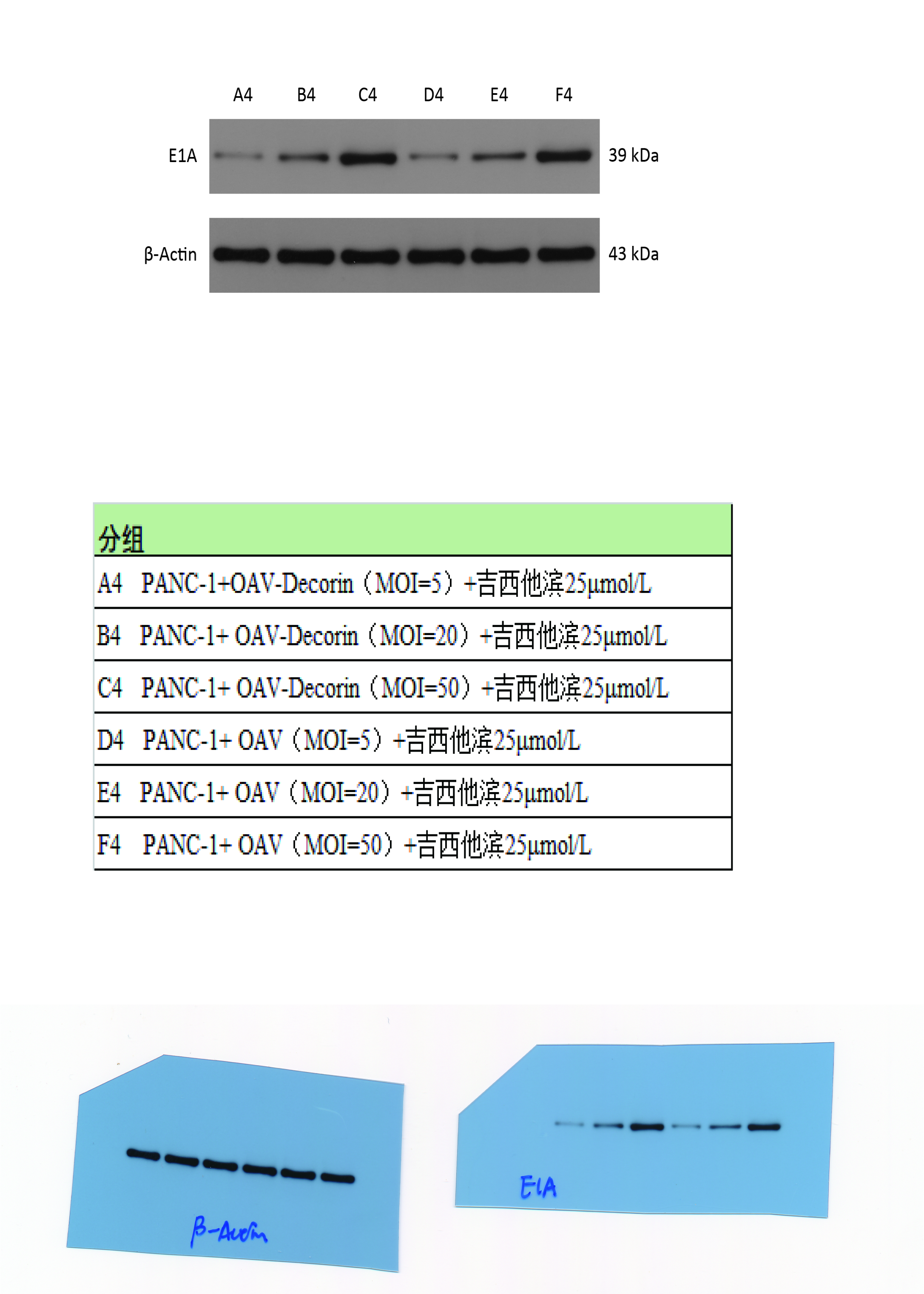

Supplement: Supplementary file 13 [file Image13.tif]

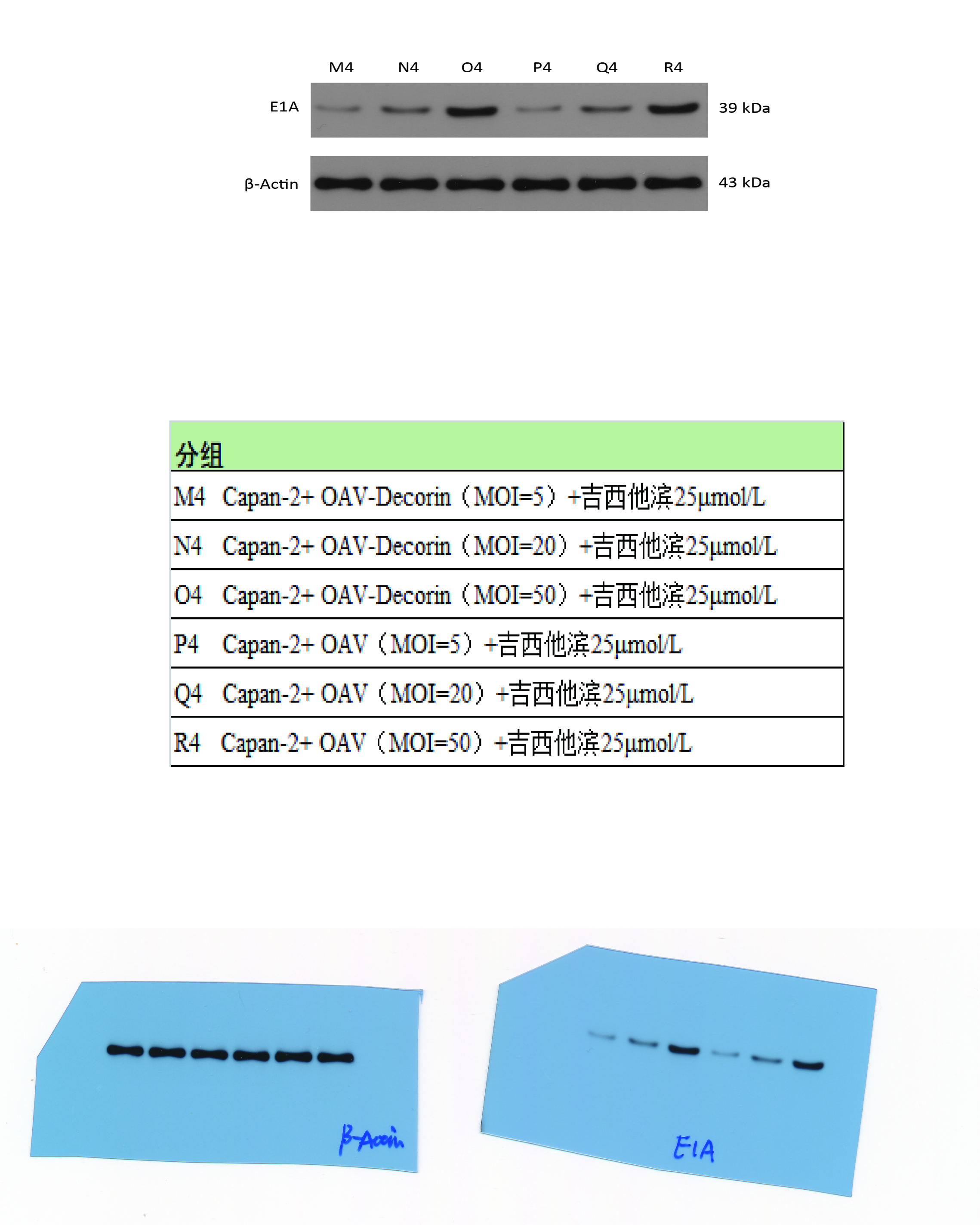

Supplement: Supplementary file 14 [file Image14.tif]

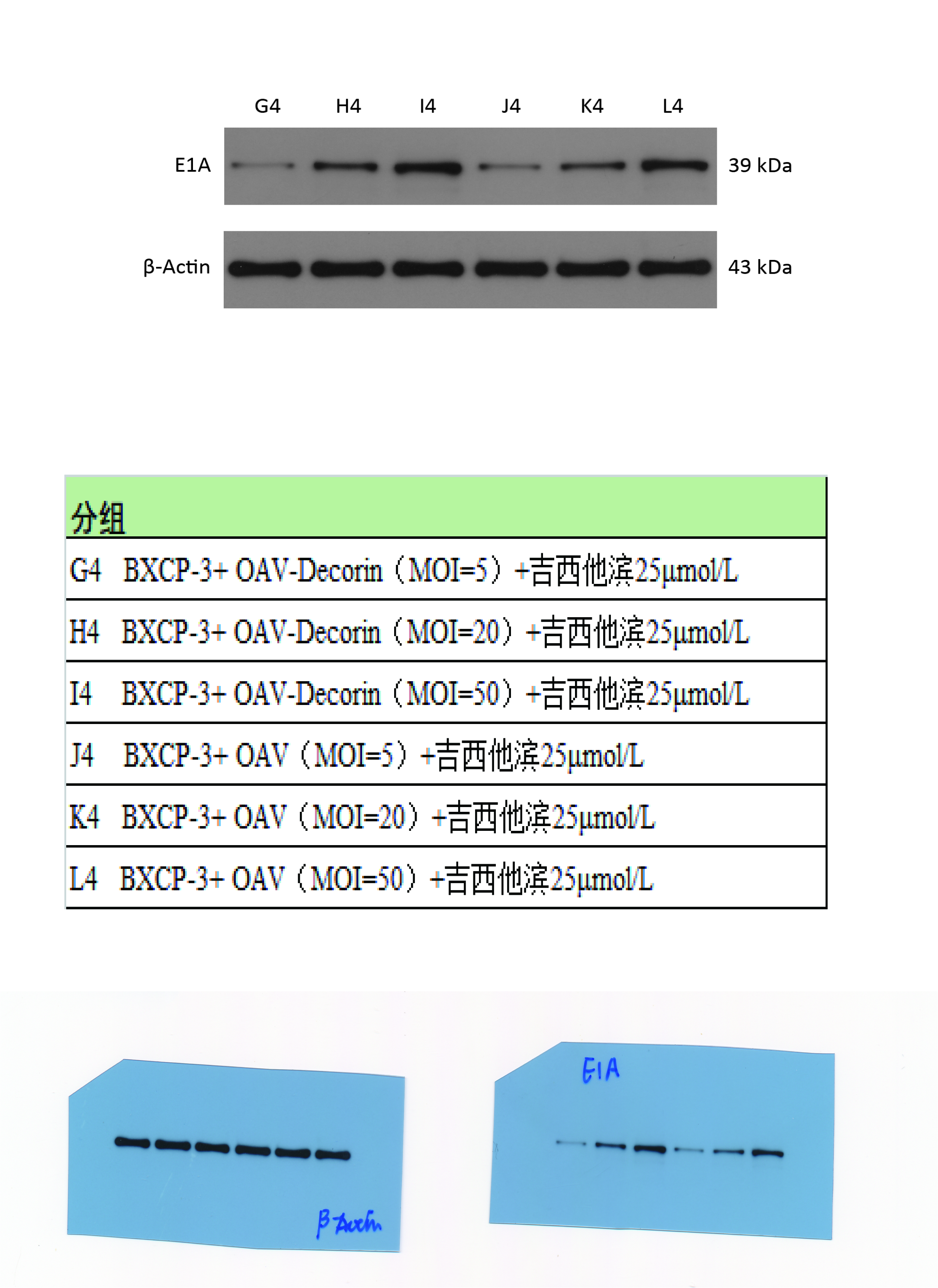

Supplement: Supplementary file 15 [file Image15.tif]
